# Molecular Analysis of Liquid-Based Cytological Specimen Using Virtually Positive Sputum with Adenocarcinoma Cells

**DOI:** 10.3390/diagnostics10020084

**Published:** 2020-02-05

**Authors:** Takeshi Nishikawa, Tomomi Fujii, Shigenobu Tatsumi, Aya Sugimoto, Yoko Sekita-Hatakeyama, Keiji Shimada, Masaharu Yamazaki, Kinta Hatakeyama, Chiho Ohbayashi

**Affiliations:** 1 Department of Diagnostic Pathology, Nara Medical University School of Medicine, 840 Shijo-cho, Kashihara, Nara 634-8521, Japan; ntakeshi@naramed-u.ac.jp (T.N.); statsu@naramed-u.ac.jp (S.T.); a_sugimoto@naramed-u.ac.jp (A.S.); yhatakeyama@naramed-u.ac.jp (Y.S.-H.); kpathol@naramed-u.ac.jp (K.H.); ohbayashi@naramed-u.ac.jp (C.O.); 2 Department of Diagnostic Pathology, Nara City Hospital, Nara 630-8305, Japan; k-shimada@nara-jadecom.jp; 3 Department of Central Clinical Laboratory, Nara Medical University Hospital, Nara 634-8521, Japan; masayama@naramed-u.ac.jp

**Keywords:** lung adenocarcinoma, EGFR mutation, liquid-based cytology, quantitative PCR

## Abstract

Liquid-based cytology (LBC) analysis of sputum is a useful diagnostic and prognostic tool for detecting lung cancer. DNA and RNA derived from lung cancer cells can be used for this diagnosis. However, the quality of cytological material is not always adequate for molecular analysis due to the effect of formalin in the commercially available fixation kits. In this study, we examined DNA and RNA extraction methods for LBC analysis with formalin fixation, using lung carcinoma cell lines and sputum. The human non-small cell lung cancer cell lines were fixed with LBC fixation reagents, such as CytoRich red preservative. Quantification of thyroid transcription factor-1 (TTF-1) and actin mRNA, epidermal growth factor receptor (EGFR) DNA in HCC827, H1975, and H1299 cells, and mutation analysis of EGFR in HCC827 and H1975 cells were performed by quantitative PCR (qPCR) and fluorescence resonance energy transfer (FRET)-based preferential homoduplex formation assay (F-PHFA) method, respectively. mRNA and DNA extracted from cell lines using RNA and/or DNA extraction kits for formalin-fixed paraffin-embedded (FFPE) fixed with various LBC solutions were efficiently detected by qPCR. The detection limit of EGFR mutations was at a rate of 5% mutated positive cells in LBC. The detection limit of the EGFR exon 19 deletion in HCC827 was detected in more than 1.5% of the positive cells in sputum. In contrast, the detection limit of the T790M/L858R mutation in H1975 was detected in more than 13% of the positive cells. We also detected EGFR mutations using next generation sequencing (NGS). The detection limit of NGS for EGFR mutation was lower than that of the F-PHFA method. Furthermore, more than 0.1% of positive cells could be cytomorphologically detected. Our results demonstrate that LBC systems are powerful tools for cytopathological and genetic analyses. However, careful attention should be paid to the incidence of false negative results in the genetic analysis of EGFR mutations detected by LBC.

## 1. Introduction

Lung cancer is the leading cause of cancer deaths in the world with more than 80% of lung cancer cases being non-small cell lung carcinoma (NSCLC). Presently, molecular targeted therapy and immunotherapy are selected in addition to conventional chemotherapy for the treatment of advanced NSCLC. Above all, molecular targeted therapy is most important for adenocarcinoma, and the importance of molecular testing for the purpose of drug adaptation including companion diagnosis has been emphasized. The molecular diagnosis and treatment of NSCLC have evolved due to the development of molecular analysis for targeted therapy including epidermal growth factor receptor (EGFR) mutations [1], anaplastic lymphoma kinase (ALK) gene rearrangements [2], and ROS proto-oncogene 1 receptor tyrosine kinase (ROS1) rearrangements [3].

Liquid-based cytology (LBC) is a useful method for the cytological diagnosis-based screening of lung cancer using sputum and bronchoalveolar lavage, because of its high precision and the availability of automatic standard systems that combine morphological assessment and molecular analyses [4,5,6,7,8,9]. The remaining fixed cells can be used for molecular analysis by extracting nucleic acids such as DNA or RNA for advanced studies. Formaldehyde-added fixation for LBC presents a difficult challenge for molecular-based testing, because of extensive crosslinking between cellular proteins and/or nucleic acids and nucleic acid degradation which could disrupt the polymerase chain reaction DNA amplification. Our previous studies showed that the detectable amount of RNA derived from LBC fixed cells using 55% methanol/0.4% formaldehyde was significantly decreased compared to that from unfixed cells or cells fixed without formaldehyde [10]. A recent study on LBC specimens of lung cancer cells has shown that genomic DNA damage can be reduced by extracting DNA using the extraction protocol for Formalin fixed paraffin embedded (FFPE) samples [11].

In this study, we examined how the stability and detection sensitivity of extracted DNA and RNA differ for several LBC systems. Several NSCLC cell lines with/without EGFR mutation were fixed using four different methanol-based fixative solvents, 55% methanol, and 55% methanol including 0.4% formaldehyde, PreservCyt Solution for ThinPrep (TP) (for non-gynecologic purposes), or BD CytoRich red preservative (CR). We selected the EGFR exon 19 region and EGFR mutation for the evaluation of DNA, and thyroid transcription factor-1 (TTF-1) mRNA, which is one of the markers of lung adenocarcinoma. The sensitivity between genetic detection of EGFR mutation and cytological analysis of LBC specimens from sputum was examined.

## 2. Materials and Methods

### 2.1. Cell Lines

Human cancer cell lines were used in this study following procedures that were in accordance with ethical standards formulated in the Declaration of Helsinki (253-6, 1 April 2018).

The human NSCLC (adenocarcinoma) cell lines H1299, HCC827, and H1975 were purchased from the American Type Culture Collection (Manassas, VA, USA). These cell lines were cultured in RPMI 1640 media supplemented with 10% fetal bovine serum and 50 units/mL penicillin-streptomycin.

### 2.2. Cell Treatment

H1299, HCC827, or H1975 cells were collected and counted using the cell counter Vi-Cell XR 2.04 (Beckman Coulter, Tokyo, Japan). Cells were diluted to 1 × 10^5^ cells/mL in Phosphate- buffered saline (PBS) and further diluted by 10 to 10,000 times and 5, 10, and 25 cells were directly counted. The diluted cells were fixed for 48 h or 2 weeks in 55% methanol, a 55% methanol/0.4% formaldehyde solution, PreservCyt Solution for ThinPrep (TP: contains 55% methanol) (for non-gynecologic purposes) (Hologic Japan, Inc., Tokyo, Japan), or CytoRich red preservative (CR: containing 23% isopropanol, 10% methanol, 0.4% formaldehyde and 6.7% ethylene glycol) (Becton Dickinson and Company, Franklin Lakes, NJ, USA).

### 2.3. Immunocytochemistry

LBC slides were prepared for immunocytochemistry analysis by fixing H1299 cells with CR or TP and stained with antibodies against TTF-1 (SPT24) (Leica, Wetzlar, Germany). The TTF-1 antibody was used at a dilution of 1:200, and the reaction mixture was incubated at room temperature for 60 min. Immunoreactions were visualized with a Histofine Simple Stain™ MAX PO (MULTI) kit (Nichirei, Tokyo, Japan).

### 2.4. Extraction of DNA and RNA and qPCR

Fixed cells were collected and the DNA or total RNA were extracted using a QIAamp DNA Mini kit or QIAamp DNA FFPE Tissue Kit (Qiagen, Venlo, The Netherlands) for DNA, and miRNeasy Mini kit or miRNeasy FFPE Kit (Qiagen) for total RNA. For reverse transcription, first-strand cDNA was synthesized from 0.5 g total RNA using PrimeScript RT Master Mix (Perfect Real Time, TaKaRa, Shiga, Japan). qPCR was performed for DNA and cDNA using SYBR Premix Ex Taq II (TliRNaseH Plus, TaKaRa) and TaqMan Universal PCR Master Mix II (Applied Biosystems, Foster City, CA, USA), respectively. Templates were initially denatured at 95 °C for 30 s and targets were amplified for 35–45 PCR cycles at 60 °C. 

The following qPCR primers were used:

Actin sense 5′-CTCTTCCAGCCTTCCTTCCT-3′
Actin antisense 5′-AGCACTGTGTTGGCGTACAG-3′
EGFR sense 5′-GCAATATCAGCCTTAGGTGCGGCT-3′
EGFR antisense 5′-CATAGAAAGTGAACATTTAGGATGTG-3′
TTF-1 sense 5′-GGACGACTTGGAACGGTTTA-3′
TTF-1 antisense 5′-TTGTCTGCACTCTCAATGCC-3′

### 2.5. Epidermal Growth Factor Receptor (EGFR) Mutation Detection

F-PHFA was used for the detection of EGFR mutation according to the manufacturer’s instructions. Briefly, 100 ng of genomic DNA was used to perform PCR containing a customized primer mix. Products were validated by agarose gel electrophoresis and EGFR mutation detected using the F-PHFA reaction kit and CFX96 Real-Time System (Bio-Rad, Hercules, CA, USA).

For NGS analysis we used AmpliSeq for Illumina Cancer Hotspot Panel v2 (Illumina, San Diego, CA, USA) according to the manufacturer’s instructions. The detection and analysis were performed by Miniseq and VariantStudio Software (Illumina).

### 2.6. Specimens of Sputum and Cytological Evaluation

This study received approval from the ethics committee of Nara Medical University (IRB1614), and informed consent was obtained from all patients. Twenty sputum samples were obtained from patients with bronchitis, pneumonia, and lung cancer. Negative cytological specimens of sputum were collected. For a precise cell count, 0.5% dithiothreitol (DTT) was added for the mucolytic treatment of sputum. HCC827 or H1975 cells of 100, 250, 500, 1000, and 2000 cells and saline (+0.5% DTT) were added to 100 mg of sputum. They were mixed using a syringe and the pellet was obtained by centrifugation (800× *g*, 5 min). 100 mL of CR was added to the cell pellet and fixed for 48 h. Cytological preparation was performed according to the manufacturer’s recommendations. Briefly, 8 mL of cell suspension was diluted with 4 mL of BD PrepStain Density Reagent, and the epithelial cells were separated from other cells by density gradient centrifugation (200× *g*, 2 min).

Cells were resuspended in purified water and bound to a SurePath PreCoat slide and visualized by Papanicolaou stain.

### 2.7. Statistical Analysis

Statistical analysis was performed with Prism 6.0 (GraphPad Software, San Diego, CA, USA) using the *t*-test for comparison between two groups. Data are presented as the mean ± SEM. Results with *p* < 0.05 were considered significant.

## 3. Results

### 3.1. Efficiency of DNA and RNA Extraction

**Figure 1:**
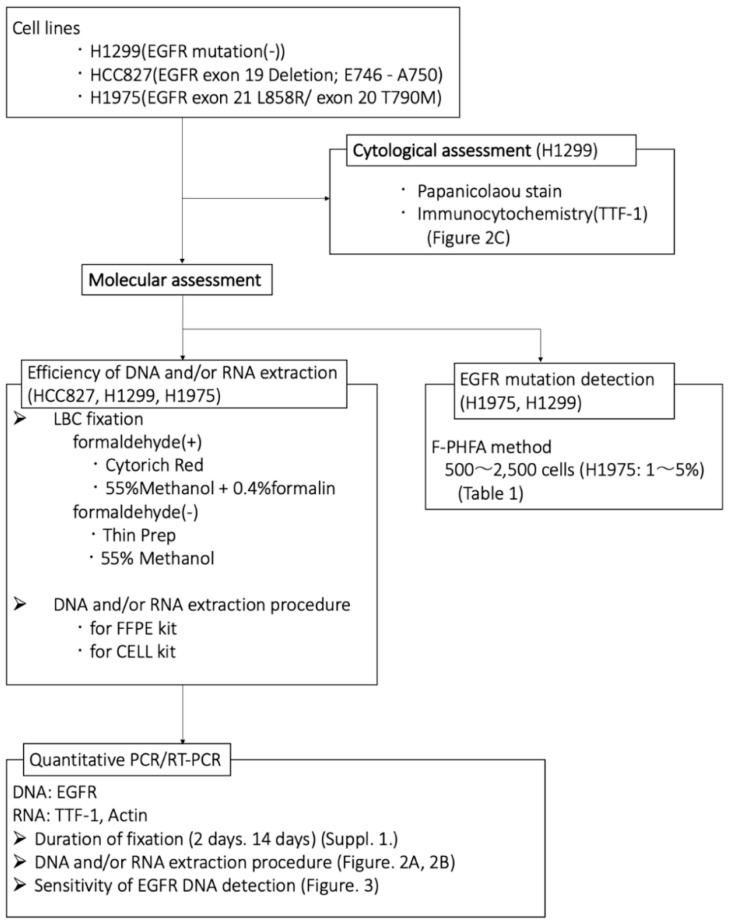
The scheme for DNA and RNA sample preparations using three adenocarcinoma cell lines. HCC827 and H1975 have epidermal growth factor receptor (EGFR) mutations.

**Figure 2:**
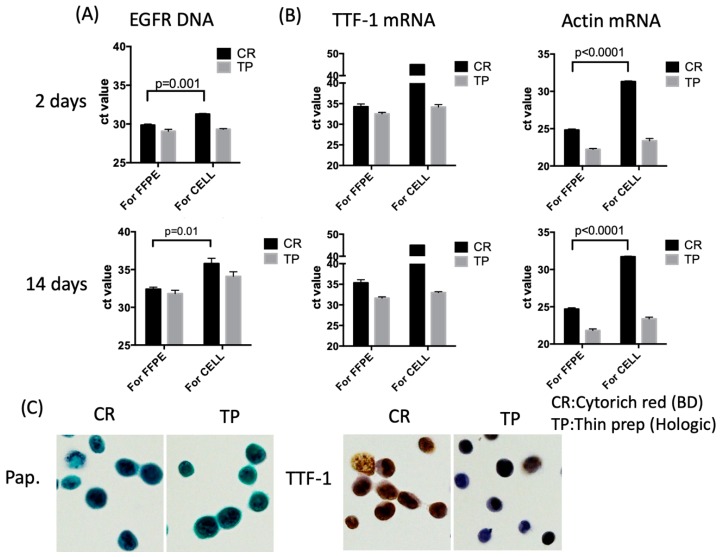
(**A**) Quantitative PCR for EGFR (exon 19 region) in H1975 cells; (**B**) Quantitative RT-PCR for thyroid transcription factor-1 (TTF-1) and actin mRNA in H1975 cells. (**A**,**B**) H1975 cells fixed with CytoRich (CR) or ThinPrep (TP) for 2 or 14 days; (**C**) Papanicolaou stain and immunocytochemistry for H1299 cells fixed with CR or TP for 5 days (×400, respectively).

Figure 1 shows the scheme for DNA and RNA sample preparations. We first evaluated the efficiency of DNA and RNA extraction from H1975 and HCC827 cells after fixation for two and 14 days, from the Threshold Cycle (*C*_t_ value) of EGFR DNA and TTF-1 and/or actin mRNA. The extraction efficiency of DNA after fixation for 14 days showed increased *C*_t_ value in both CR and TP fixed cells. On the other hand, there was no significant difference in the extraction efficiency of RNA between cells that were fixed for two and 14 days (Figure S1). EGFR DNA was effectively detected from fixed cells using both CR and TP fixation, but the extraction efficiency was significantly improved in the cells fixed with CR using the FFPE extraction kit. TTF-1 mRNA could not be detected in the cells fixed using CR and extracted using the above kit. The extraction efficiency of mRNA was considerably improved using the FFPE extraction kit. Actin mRNA was also detectable in all samples, but the extraction efficiency was considerably improved in the cells which were fixed using CR and extracted using the FFPE extraction kit (Figure 2A,B and Figure S2).

### 3.2. Immunocytochemistry of Liquid-Based Cytology (LBC)

We examined the immunoreactivity of cells fixed with CR and TP. H1299 cells were fixed for 5 days with CR or TP and then immunostained using TTF-1 antibodies. Cells fixed with CR and TP showed good staining patterns without any differences (Figure 2C).

### 3.3. DNA Detection Sensitivity

**Figure 3:**
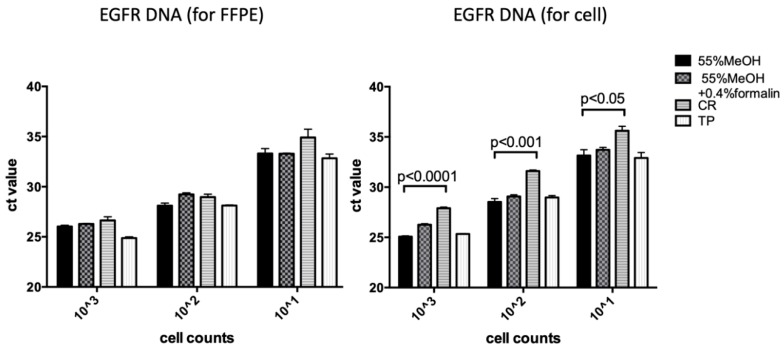
Detection sensitivity of quantitative PCR for EGFR DNA in H1299 cells. Cells were fixed with 55% methanol, 55% methanol + 0.4% formalin, CR, or TP.

H1299 and HCC827 cells were prepared at cell counts of 10, 100, and 1000 cells for DNA detection sensitivity examination using the quantitative PCR (qPCR) method. The cells were fixed for five days in 55% methanol, 55% methanol + 0.4% formaldehyde, CR, and TP, and then DNA was extracted. No significant difference was found in the *C*_t_ values among four kinds of DNA samples extracted using the FFPE kit (Figure 3, Figure S3; left graph). In contrast, DNA fixed with CR and extracted using the DNA extraction kit for cells had higher *C*_t_ values than the other samples (Figure 3, Figure S3; right graph). These results suggest that CR might contain additional components that interfere with DNA detection. The formalin in the DNA extraction kits might also have an effect, which could be improved using the FFPE kit.

### 3.4. Detection of EGFR Mutation

We conducted the detection of EGFR mutations to confirm the application of the molecular targeted therapy for NSCLC such as with the EGFR-tyrosine kinase inhibitor. However, NSCLC is typically diagnosed in a carcinoma tissues obtained by biopsy or surgery. This diagnosis is usually possible if the biopsy or surgical tissue material contains more than 10% of tumor cells. We examined EGFR mutations using H1975 and H1299 cells. H1975 (T790M, L858R+) and H1299 (wild type) cells were mixed according to the ratio shown in Table 1. EGFR was detected by fixing the mixed cells using CR and the DNA was extracted using the FFPE kit after fixation for five days. The total cell count was 500–2500, and EGFR mutation could be detected in samples that included 5% of mutation-positive cells (Table 1).

### 3.5. Detection of EGFR DNA Mutation from Sputum

**Figure 4:**
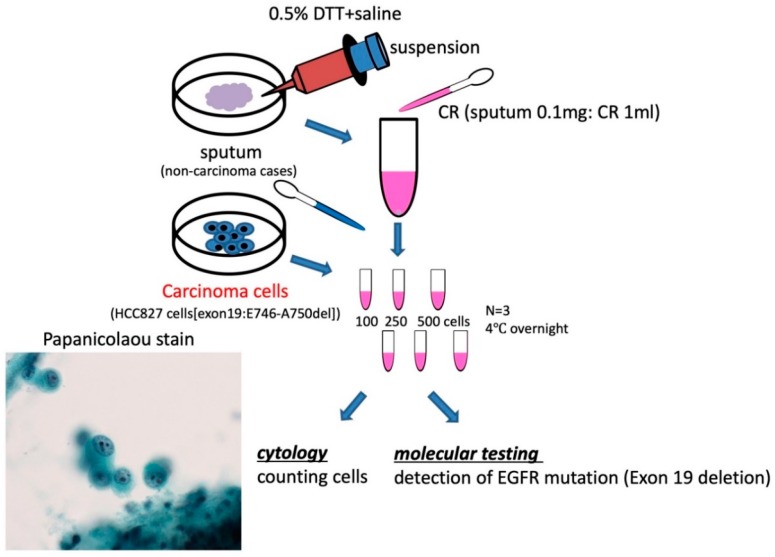
The treatment procedure for virtually positive sputum specimen of LBC (×400). (see materials and methods).

**Figure 5:**
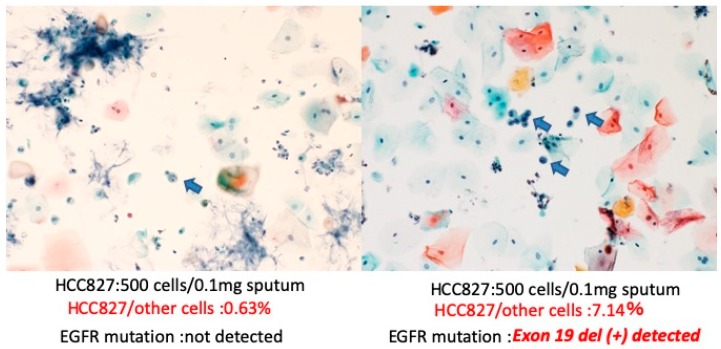
Papanicolaou stain for sputum LBC specimen containing HCC827 cells (×200). The **left** panel shows cytology specimen of case 3 including 500 cells of HCC827/0.1 mg sputum; The **right** panel shows cytology specimen of case 1 including 500 cells of HCC827/0.1 mg sputum. Arrows indicate HCC827 cells in the sputum.

To confirm the utility of molecular testing of EGFR mutation in the sputum for lung cancer, sputum containing a known number of EGFR mutation-positive cells were artificially prepared, that is, HCC827 cells (exon19:E746-A750del) were mixed with cancer-negative sputum, and EGFR mutations were detected. Figure 4 shows a flowchart of the sputum specimen treatment. Briefly, 100, 250, or 500 HCC827 cells were mixed in sputum and fixed in CR for two days. The positive cells were counted and EGFR mutation detected in the fluorescence resonance energy transfer (FRET)-based preferential homoduplex formation assay (F-PHFA) method. In case 1, both cytology and genetic EGFR mutation could be detected in 1.43% carcinoma cells (total of 7000 cells). In case 2, both cytology and genetic EGFR mutation could be detected in 2.17% carcinoma cells in a total of 23,000 cells. However, in cases 3 and 4, more than 0.1% carcinoma cells (total of 80,000 and 172,000 cells respectively) were detectable in cytology, but were not detectable in genetic analysis (Figure 5 and Table 2). These results suggest that false-negative results may be observed in less than 1% of cancer cells in the detection of EGFR mutation using the LBC specimen.

Next, to confirm our results, we checked the genetic alteration of clinical assessments. H1975 cells (T790M, L858R) were mixed with cells from cancer-negative sputum, and EGFR mutations were detected. Thirteen cases were investigated for drug-resistant mutations following the same procedure (Figure 4). Briefly, 500, 1000, or 2000 H1975 cells were mixed in sputum and fixed in CR for two days. The positive cells were counted and EGFR mutation detected by the F-PHFA method. Unexpectedly, genetic EGFR mutations (T790M, L858R) were detected in at least 12% of carcinoma cells (Table 3). These detection limits were lower than exon19 deletion. These results suggest that each carcinoma cell with different mutation results in different limits in genetic analysis.

We used another tool, next generation sequencing (NGS), for the detection of EGFR mutation. For the detection of EGFR mutation, the target panel, i.e., AmpliSeq for Illumina Cancer Hotspot Panel v2 was used. Eleven cases for EGFR exon19 deletion and five cases of T790M/L858R mutation were tested. For the exon 19 deletion, ten of eleven cases were detected by the F-PHFA method, and eight of the eleven cases were detected by NGS. On the other hand, for T790M/L858R mutation, three of nine cases were detected by F-PHFA. Two of five cases were detected by NGS. The detection limit of EGFR mutation was lower in NGS than in F-PHFA, and the concordance rate between the F-PHFA method and NGS was 82% for exon 19 deletion and 80% for the T790M/L858R mutation (Table 4).

## 4. Discussion

Some somatic mutations or chromosomal alterations induce protein kinase activation and tumorigenesis [12]. In the past decade, effective approaches in cancer therapy have been explored such as inhibition of activated protein kinases via the use of specific targeted small molecules and/or antibody-based drugs [13,14,15]. EGFR is a transmembrane receptor that interacts with different ligands, showing cell growth effect via tyrosine kinase signaling on different cell types. The previous study identified EGFR tyrosine kinase domain mutations were identified and suggested that the role of EGFR as a growth factor receptor might be essential in tumor growth via tyrosine kinase signaling activity [1,16,17]. The current therapeutic strategy for NSCLC is molecular targeted therapy such as the use of EGFR-tyrosine kinase inhibitors (EGFR-TKIs) for NSCLC with EGFR gene mutation [18,19,20]. Molecular testing for EGFR mutations is one of the most important tools currently being used for the diagnosis and therapeutic management of NSCLC [21,22]. There is a race difference in frequency of the EGFR mutation, whereby it is more common in Asia than in Europe and America, and half of cases of the NSCLC cases are positive for EGFR mutation in Japan [23].

The continuous treatment using EGFR-TKI for EGFR-mutant NSCLC develops resistance to EGFR-TKIs, due to the acquired secondary EGFR mutations such as T790M on exon 20 of the EGFR gene [19,24,25,26]. Re-biopsy is desirable for the early detection of tumor recurrence and proliferative reactivation of carcinoma by acquired drug resistance [19,27,28]. However, this is not practical because of the high invasiveness involved. The sputum for diagnostic-cytology is a simple and easy technique, however, it cannot precisely determine the number of degenerate carcinoma cells. The combination of cytology and genetic screening using the LBC specimen of sputum is an objective and excellent technique for the diagnosis of NSCLC.

Sputum is a bodily fluid that can be acquired easily in a noninvasive manner and contains exfoliated epithelial cells derived from the bronchus. In cytology of post-bronchoscopy sputum samples, 12.5% of cases were diagnosed with suspicious malignancy [29]. To detect malignant cells and specific gene mutations, high quality molecular testing is required for developing of the diagnosis of NSCLC. Several advanced molecular techniques have been developed using sputum in the prognosis and/or the sensitive detection of lung cancer [30]. Recent studies on molecular testing with NSCLC have shown that small nucleolar RNAs are readily detectable in sputum [31]. Some useful molecular biomarkers such as microRNAs from sputum have also been identified [32,33,34,35,36,37,38]. Cytology and molecular testing using LBC specimen of the pleural effusion is one of the powerful tools in progressive or metastatic carcinoma [39,40,41]. However, efficient molecular testing techniques to detect common gene mutations in EGFR using LBC specimens from the sputum of patients with lung cancer, have not yet been proposed. In our recent study, we showed that 1.43% of malignant EGFR mutation cells could be detected in sputum LBC specimens containing 7000 cells/mL. It can also be a specific method for specimen preparation designed for BD SurePath, despite only 0.1% of carcinoma cells being detectable in LBC for sputum. The commercial preparation reagent for BD SurePath contributes to selecting cells by partially removing non-diagnostic debris and excessive inflammatory cells from the sample. EGFR mutation is detectable if the carcinoma cell content is more than 1.5%, but cytology maybe more sensitive at a lower cellular content. In our recent study, TP showed lower Ct values than CR. However, we routinely use CR for LBC. In Japan, CR is widely used for LBC in the clinical laboratory. Therefore, we wanted to know whether residual materials fixed with CR could be effectively used for molecular testing effectively after cytological testing. We used CR for the detection of EGFR mutation from sputum with lung carcinoma cell lines.

Molecular testing of the sputum is very useful for evaluating inflammatory lung disease as well as diagnosing lung cancer [42,43,44]. The detection of free DNA in the sputum reflects the presence of carcinoma cells and inflammation, similar to that seen in cellular DNA [45]. However, sputum may include a lot of inflammatory cells which could be problematic for detecting DNA derived from carcinoma cells. Detecting DNA from a cancer-specific gene, such as the EGFR mutant allele, may become difficult if many inflammatory cells are included in the sputum. For example, our case 2 sample included a lot of inflammatory cells and the EGFR mutation could not be detected, even when testing 1% of the cells. It would be useful to identify a target gene that can be used as a tool to differentiate inflammatory lung disease from lung cancer. We did not succeed in developing a method to separate inflammatory cells from epithelial cells. Future studies should search for a useful target gene in free DNA and cellular DNA as a method to differentiate inflammation from carcinoma.

The exhaustive detection of a somatic gene mutation related to carcinoma is performed using NGS and other useful molecular analysis tools. In lung cancer, it is very useful to perform molecular testing using cellular and free DNA to differentiate primary lung cancer from a metastatic tumor, especially if tissue sampling is not possible. In this study, we performed an NGS analysis for EGFR mutations in the sputum. EGFR mutation detection using the prepared positive sputum sample was detectable in NGS, but less sensitive than F-PHFA. NGS requires a sufficient amount of high-quality DNA to detect many gene mutations. Therefore, the sensitivity is strict compared to the F-PHFA method of the EGFR-specific detection method.

Sputum may be used as one of the non-invasive tests for advanced stage lung cancer using LBC, which is a non-invasive and simple method that can confirm the presence of tumor cells. Therefore, the adaptation of LBC samples to NGS should be investigated based on a sufficient understanding of the detection sensitivity of gene mutations. In the future, we plan to use NGS analysis to search for variable cancer-specific genes using the DNA derived from sputum.

The important and practical outcome of this study is that the proposed method is capable to perform genetic analysis when sputum containing tumor cells, and to embody the sensitivity of detecting tumor cells. Therefore, confirmation experiments using clinical materials containing tumor cells with EGFR mutations in sputum are necessary. However, adenocarcinoma appears only in advanced cancer stages in sputum. During the study period, sputum derived from patients with lung adenocarcinoma contained carcinoma cells in some cases, but all cases were EGFR-negative based on histological diagnosis. Therefore, in this study, we used a virtually positive sputum to study only the known cell number. Additional experiments using clinical samples will be necessary in the future. In addition, at present, no gene abnormality effective for companion diagnosis has been proposed for squamous cell carcinoma, but it will be widely used in the future if the gene analysis of tumor cells in sputum becomes useful in lung cancer cases other than adenocarcinoma. Sputum is a secretion fluid containing many proteolytic enzymes and is a very strict material to be used for genetic testing. In this study, we demonstrated that solubilization of sputum by DTT and conversion to LBC enable efficient simultaneous cytology and genetic analysis. Thus, our results provide valuable data in the field of pathological cytology.

## 5. Conclusions

LBC is an excellent method for cytology where carcinoma cells can be collected effectively. However, it is difficult to perform molecular testing on the remaining sample because of formaldehyde content in the fixative solution to make high quality specimen and good staining. Our recent study proposed a useful method that can extract DNA and RNA efficiently from LBC with a fixative solution containing formalin. EGFR mutation detection was possible from 5% of lung adenocarcinoma cell lines. The sputum is a useful specimen that can be easily collected from lung cancer patients. The cell count and detection from a sputum specimen can be facilitated by the addition of DTT for mucolysis. In this study, EGFR mutation detection was possible if 1.5% of carcinoma cells were included, but detection was also possible by cytology if the cancer cell content was 0.1% since background cells such as inflammatory cells and debris could be removed from the specimen.

Attention must be paid to false-negative results when conducting EGFR mutation detection with a sample containing cells other than tumor cells.

Hence, a method for the removal of background cells from sputum LBC specimens must be explored to improve the sensitivity and efficiency of EGFR mutation detection. 

In the future, we plan to demonstrate practical verification because we could not obtain real clinical materials with EGFR mutation in this study. Our studies provide important fundamental data for bridging morphology and genetic analysis for pathological and cytological experts.

## Figures and Tables

**Table 1:** Detection of EGFR mutation.

	Cell Counts (Percentage of Mutant Cells)				
**H1975 (T790M, L858R)**	5	10	25	25	25
**H1299 (wild type)**	495	490	475	975	2475
**Total counts**	500 (1%)	500 (2%)	500 (5%)	1000 (2.5%)	2500 (1%)
	N.D.	N.D.	T790M(+), L858R(+)	N.D.	N.D.

N.D.: not detected.

**Table 2:** Cell counts and EGFR mutation detection in the sputum (1).

	Clinical Diagnosis/Symptom	Cell Counts		LBC (Cytology and Molecular Testing)				
		Total Cell Counts	HCC827 (% of HCC827 Cells)	Total HCC827 Cell Counts (All Fields)	Epithelial Cells (/HPF)	Other Cells * (/HPF)	DNA Amount (μg)	EGFR (Ex 19 del)
**Case 1**	Bronchial asthma	7000	100 (1.43)	31	76	31	9.81 × 10^−3^	(+)
			250 (3.57)	89	88	39	19.1 × 10^−3^	(+)
			500 (7.14)	192	79	34	20.3 × 10^−3^	(+)
**Case 2**	Bronchiectasis	23,000	100 (0,43)	3	93	43	1.86	(−)
	Bloody sputum		250 (1.09)	32	96	56	1.59	(−)
			500 (2.17)	54	101	56	1.34	(+)
**Case 3**	Bronchiectasis	80,000	100 (0.13)	40	171	5	2.69	(−)
	Bloody sputum		250 (0.31)	37	159	4	2.87	(−)
			500 (0.63)	89	162	3	3.81	(−)
**Case 4**	Emphysema	172,000	100 (0.06)	0	116	5	2.63	(−)
	Pneumonia		250 (0.15)	9	123	4	4.39	(−)
			500 (0.29)	6	110	5	4.28	(−)

*: lymphocytes and neutrophils.

**Table 3:** Cell counts and EGFR mutation detection in the sputum (2).

	Cell Line	Cell Counts		LBC (Cytology and Molecular Testing)			
		Total Cell Counts	HCC827/H1975 (% of Cancer Cells)	Total Cancer Cell Counts (All Fields)	DNA Amount (μg)	EGFR (T790M)	EGFR (Ex 19 del)
**Case 5**	HCC827	8000	500 (6.25)	81	1.17		(+)
	H1975		500 (6.25)	36	0.93	(−)	
	H1975		1000 (12.5)	85	0.82	(−)	
	H1975		2000 (25.0)	445	0.17	(+)	
**Case 6**	HCC827	10,000	500 (5.00)	89	1.70		(+)
	H1975		500 (5.00)	32	1.96	(−)	
	H1975		1000 (10.0)	69	1.65	(−)	
	H1975		2000 (20.0)	371	1.26	(−)	
**Case 7**	HCC827	15,500	500 (3.23)	97	0.98		(+)
	H1975		500 (3.23)	41	0.83	(−)	
	H1975		1000 (6.45)	129	0.58	(−)	
	H1975		2000 (12.9)	330	0.54	(+)	
**Case 8**	HCC827	33,500	500 (1.49)	15	4.19		(+)
	H1975		500 (1.49)	29	2.10	(−)	
**Case 9**	HCC827	9000	500 (5.56)	10	0.78		(+)
	H1975		500 (5.56)	7	0.77	(−)	
**Case 10**	HCC827	27,000	500 (1.85)	14	1.22		(+)
	H1975		500 (1.85)	22	1.11	(−)	
**Case 11**	HCC827	15,500	500 (3.23)	24	2.52		(+)
	H1975		500 (3.23)	47	2.61	(−)	
**Case 12**	HCC827	15,000	500 (3.33)	52	4.47		(−)
	H1975		500 (3.33)	48	3.11	(−)	
	H1975		1000 (6.67)	122	2.34	(−)	
	H1975		2000 (13.3)	367	2.15	(−)	
**Case 13**	HCC827	20,000	500 (2.50)	11	4.50		(+)
**Case 14**	HCC827	38,000	500 (1.85)	8	1.50		(+)
**Case 15**	HCC827	8500	500 (5.88)	45	0.83		(+)
**Case 16**	H1975	14,500	2000 (13.8)	369	0.56	(+)	
**Case 17**	H1975	19,000	2000 (10.5)	256	3.24	(−)	
**Case 18**	H1975	17,000	2000 (11.8)	164	7.53	(−)	
**Case 19**	H1975	18,500	2000 (10.8)	196	1.95	(−)	
**Case 20**	H1975	13,000	2000 (15.4)	186	1.13	(−)	

**Table 4:** Cell counts and EGFR mutation detection in the sputum (3).

	Cell Line	Cell Counts		LBC (Cytology and Molecular Testing)			NGS
		Total Cell Counts	HCC827/H1975 (% of Cancer Cells)	Total Cancer Cell Counts (All Fields)	DNA Amount (μg)	EGFR Mutation (F-PHFA)	Cancer Hot Spot Panel v2
**Case 5**	HCC827	8000	500 (6.25)	81	1.17	Ex19 del +	p.E746_A750del (ELREA/−)
	H1975		2000 (25.0)	445	0.17	T790M+/L858R+	T790M+/L858R+
**Case 6**	HCC827	10,000	500 (5.00)	89	1.70	Ex19 del +	(−)
	H1975		2000 (20.0)	371	1.26	(−)	(−)
**Case 7**	HCC827	15,500	500 (3.23)	97	0.98	Ex19 del +	p.E746_A750del (ELREA/−)
	H1975		2000 (12.9)	330	0.54	T790M+/L858R+	(−)
**Case 8**	HCC827	33,500	500 (1.49)	15	4.19	Ex19 del +	p.E746_A750del (ELREA/−)
**Case 9**	HCC827	9000	500 (5.56)	10	0.78	Ex19 del +	p.E746_A750del (ELREA/−)
**Case 10**	HCC827	27,000	500 (1.85)	14	1.22	Ex19 del +	p.E746_A750del (ELREA/−)
**Case 11**	HCC827	15,500	500 (3.23)	24	2.52	Ex19 del +	(−)
**Case 12**	HCC827	15,000	500 (3.33)	52	4.47	(−)	(−)
	H1975		2000 (13.3)	367	2.15	(−)	(−)
**Case 13**	HCC827	20,000	500 (2.50)	11	4.50	Ex19 del +	p.E746_A750del (ELREA/−)
**Case 14**	HCC827	38,000	500 (1.85)	8	1.50	Ex19 del +	p.E746_A750del (ELREA/−)
**Case 15**	HCC827	8500	500 (5.88)	45	0.83	Ex19 del +	p.E746_A750del (ELREA/−)
**Case 16**	H1975	14,500	2000 (13.8)	369	0.56	T790M+/L858R+	T790M+/L858R+
**Case 17**	H1975	19,000	2000 (10.5)	256	3.24	(−)	
**Case 18**	H1975	17,000	2000 (11.8)	164	7.53	(−)	
**Case 19**	H1975	18,500	2000 (10.8)	196	1.95	(−)	
**Case 20**	H1975	13,000	2000 (15.4)	186	1.13	(−)	

## Supplementary Materials

The following are available online at https://www.mdpi.com/2075-4418/10/2/84/s1.
