# Supplementary figures and images for: Molecular Analysis of Liquid-Based Cytological Specimen Using Virtually Positive Sputum with Adenocarcinoma Cells
Source: Diagnostics (Basel). 2020 Feb 5;10(2):84. doi: 10.3390/diagnostics10020084 (PMC7168204; doi:10.3390/diagnostics10020084)

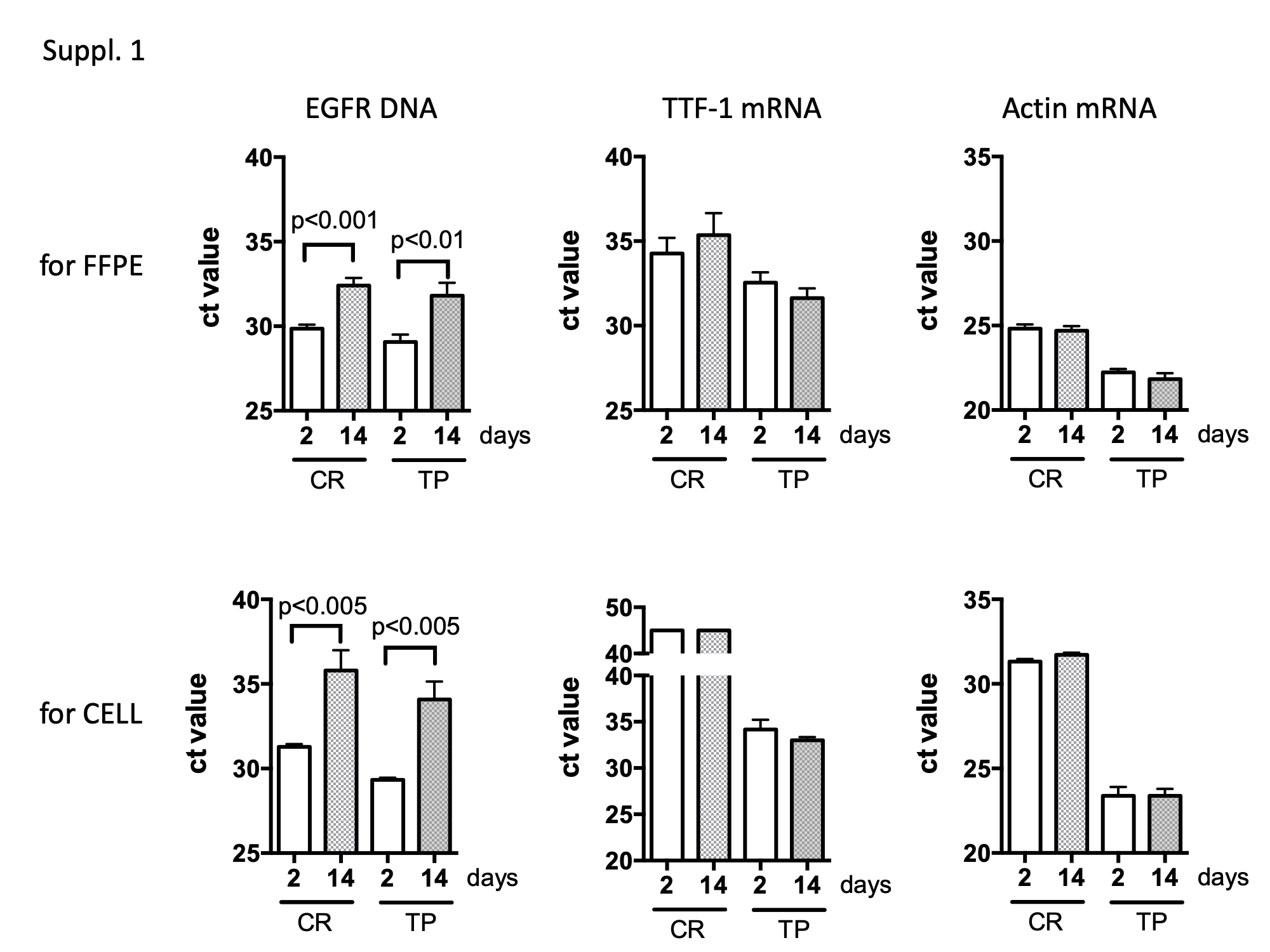

Supplement: Supplementary file 1 [file diagnostics-10-00084-s001.zip › diagnostics-703741-supplementary/Figure S1.tiff]

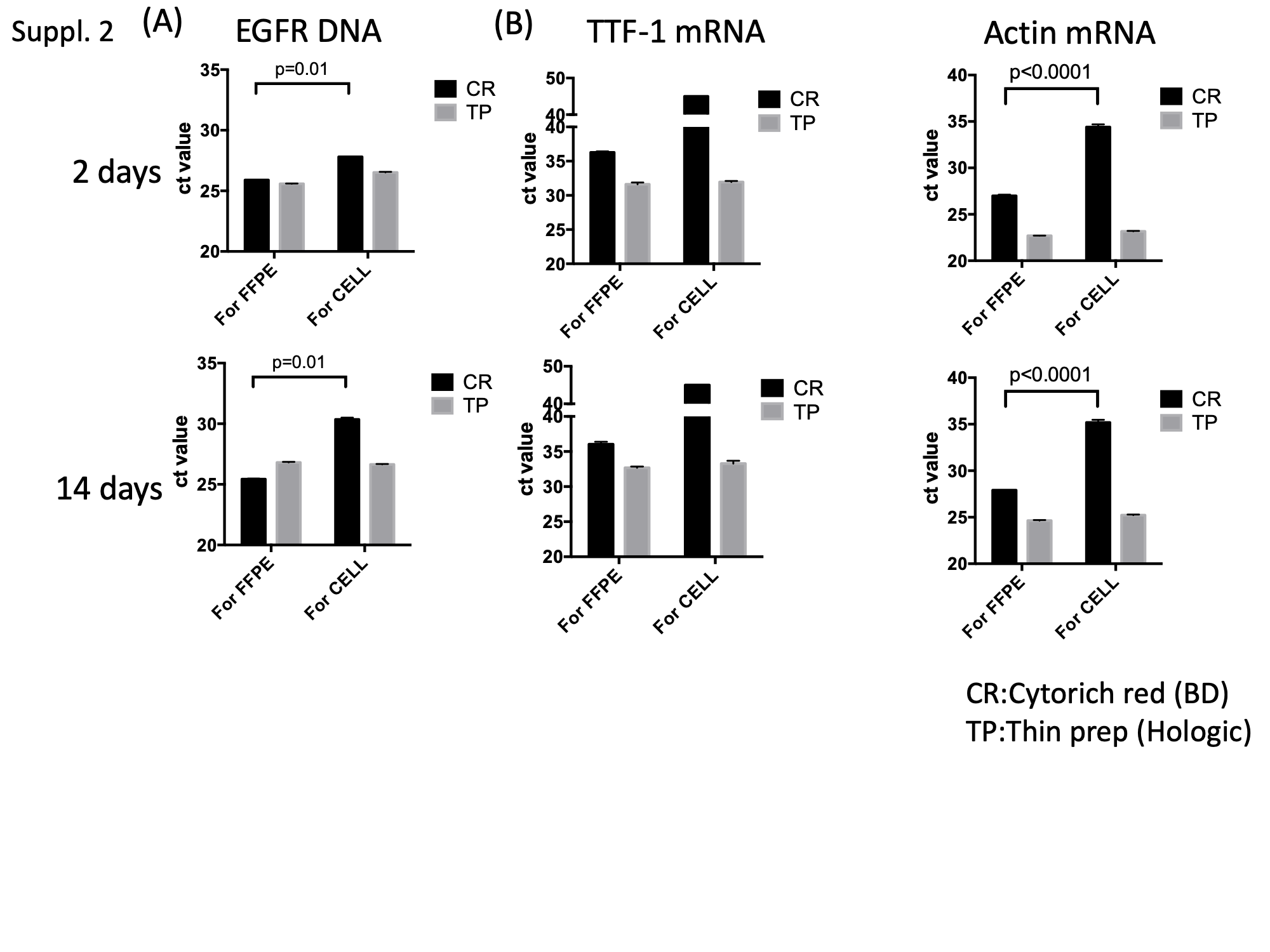

Supplement: Supplementary file 1 [file diagnostics-10-00084-s001.zip › diagnostics-703741-supplementary/Figure S2.tiff]

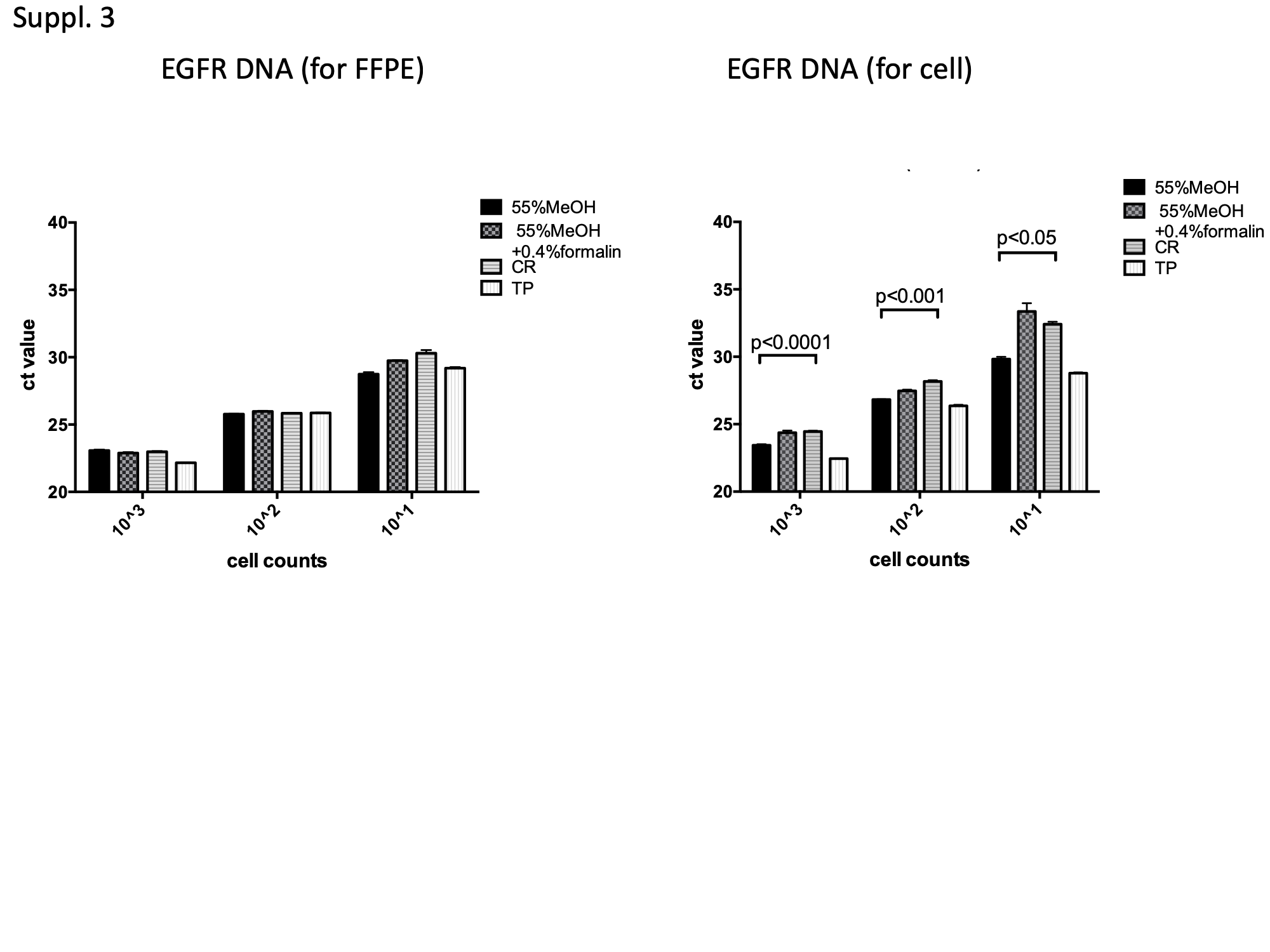

Supplement: Supplementary file 1 [file diagnostics-10-00084-s001.zip › diagnostics-703741-supplementary/Figure S3.tiff]
